# Efficacy and safety of traditional Chinese medicine adjuvant therapy for severe pneumonia: evidence mapping of the randomized controlled trials, systematic reviews, and meta-analyses

**DOI:** 10.3389/fphar.2023.1227436

**Published:** 2023-09-29

**Authors:** Kai Xie, Shengnan Guan, Hui Jing, Wenshuai Ji, Xinxin Kong, Shen Du, Mingyan Jia, Haifeng Wang

**Affiliations:** ^1^ Department of Respiratory Medicine, The First Affiliated Hospital of Henan University of Traditional Chinese Medicine, Zhengzhou, China; ^2^ Academy of Chinese Medical Sciences, Henan University of Chinese Medicine, Zhengzhou, China; ^3^ Co-construction Collaborative Innovation Center for Chinese Medicine and Respiratory Diseases by Henan & Education Ministry of P.R. China, Henan University of Chinese Medicine, Zhengzhou, China

**Keywords:** severe pneumonia, traditional Chinese medicine, evidence mapping, evidence synthesis, randomized controlled trial, systematic review

## Abstract

**Background and Objective:** Severe pneumonia is a critical respiratory disease with high mortality. There is insufficient evidence on the efficacy and safety of traditional Chinese medicine (TCM) adjuvant therapy for severe pneumonia. This study aims to identify, describe, assess, and summarize the currently available high-quality design evidence on TCM adjuvant therapy for severe pneumonia to identify evidence gaps using the evidence mapping approach.

**Methods:** Systematic searches were performed on English and Chinese online databases (PubMed, EMBASE, Cochrane Library, Web of Science, CNKI, WanFang Data, CQVIP, and SinoMed) to identify papers from inception until August 2023 for inclusion into the review. Randomized controlled trials (RCTs), systematic reviews (SRs), and meta-analyses concerning TCM adjuvant therapy for severe pneumonia or its complications in adults were included. The risk of bias in RCTs was evaluated by using the *Cochrane Handbook* ROB tool. The Assessment of Multiple Systematic Reviews 2 (AMSTAR-2), the Risk of Bias in Systematic Review (ROBIS) tool, and the Grading of Recommendations Assessment, Development and Evaluation (GRADE) system were used to assess the methodological quality, risk of bias, and evidence quality of SRs or meta-analyses, respectively. Then, a bubble plot was designed to visually display information in four dimensions.

**Results:** A total of 354 RCTs and 17 SRs or meta-analyses met the inclusion criteria. The published RCTs had several flaws, such as unreasonable design, limited sample size, insufficient attention to non-drug therapy studies and syndrome differentiation, improper selection or use of outcome indicators, and failure to provide high-quality evidence. Sixteen SRs or meta-analyses of methodological quality scored “Critically Low” confidence. Twelve SRs or meta-analyses were rated as “High Risk.” Most outcomes were rated as “Low” evidence quality. We found that TCM combined with conventional treatment could improve the clinical total effective rate and the TCM syndromes efficacy. The combined approach could also shorten mechanical ventilation time, infection control time, and length of hospital and ICU stay; significantly reduce temperature, respiratory rate, heart rate, white blood cell counts, levels of C-reactive protein, procalcitonin, blood inflammatory factors, bacteriological response, and D-dimer; decrease CPIS, APACHE II score, and PSI score; improve pulmonary imaging features, arterial blood gas indicators (including arterial oxygen pressure, arterial oxygen saturation, and oxygen index), and lung function (including forced vital capacity and forced expiratory volume in the first second) for severe pneumonia compared with conventional treatment only (*p* < 0.05). There was no significant difference in adverse reactions and incidence of adverse events (*p* > 0.05). In addition, compared with conventional treatment only, most SRs or meta-analyses concluded that TCM combined with conventional treatment was “Beneficial” or “Probably beneficial.”

**Conclusion:** TCM combined with conventional treatment had advantages in efficacy, clinical signs, laboratory results, and life quality outcomes of severe pneumonia, with no difference in safety outcomes compared with conventional treatment only. QingJin Huatan decoction is the most promising target, and Xuanbai Chengqi decoction has a “Probably beneficial” conclusion. XueBiJing injection and TanReQing injection are two commonly used Chinese herbal injections for treating severe pneumonia, and both are “Probably beneficial.” However, there was a need for multicenter RCTs with large sample sizes and high methodological quality in the future. In addition, the methodological design and quality of SRs or meta-analyses should be improved to form high-quality, evidence-based medical evidence and provide evidence for the effectiveness and safety of TCM adjuvant therapy for severe pneumonia.

## 1 Introduction

Data from the Global Burden of Disease Study reported that lower respiratory infections were the fourth most common cause of death worldwide, only after neonatal disorders, ischemic heart disease, and stroke (GBD, 2019 Diseases and Injuries Collaborators, 2020). Severe pneumonia is a common critical disease of lower respiratory infection, with high mortality, many complications, a poor prognosis, and a heavy economic burden (Welte et al., 2012). Pneumonia accounts for 78% of infection-related deaths in the United States (Welte et al., 2012; Martin-Loeches et al., 2022).

Currently, the main measures of conventional treatment for severe pneumonia are anti-infection, mechanical ventilation, application of glucocorticoids, and so on (Jackson et al., 2020). Despite the continuous progress of modern medical technology, high mortality rates have always accompanied severe pneumonia, and treatment remains challenging. Therefore, it is essential to explore more effective treatment approaches. Conventional treatment is routinely used in developed nations and in Eastern countries, where traditional Chinese medicine (TCM) is frequently used by a selection of patients as an alternative and complement to conventional treatment. Many published reports have shown that TCM could be effectively used in the management of acute and critical illnesses (Wang et al., 2017; Wang et al., 2018). Nonetheless, the efficacy and safety of TCM adjuvant therapy for severe pneumonia require more evidence.

Randomized controlled trials (RCTs) are the most reliable evidence to evaluate the efficacy and safety of interventions (Woodbridge et al., 2018). Systematic reviews (SRs) or meta-analyses are critical evidence synthesis methods. According to the evidence classification system, the best evidence for evidence-based medicine originates from RCTs and associated SRs or meta-analyses (Zheng et al., 2020). Currently, several RCTs on TCM adjuvant therapy for severe pneumonia have been completed. Additionally, an increasing number of SRs or meta-analyses have been conducted. RCTs, SRs, and meta-analyses focus on a single clinical question, and the reliability, methodology and reporting quality of these studies tends to affect the judgment of the intervention results (Hoffmann et al., 2017; Bonetti et al., 2020), and incomplete reports may impact the proper choice of intervention measures (Hoffmann et al., 2017). Therefore, it is necessary to comprehensively and systematically collect and analyze current high-quality design studies to further clarify the clinical efficacy of TCM adjuvant therapy for severe pneumonia.

Evidence mapping is a synthetic evidence-based research methodology that is increasingly used to systematically and rapidly identify, assess, synthesize, and display existing evidence to provide directions for future studies (Sun et al., 2020; Yang et al., 2021). Evidence mapping provides a visual overview of existing evidence in a certain research field and clarifies the characteristics of the studies in this field from multiple dimensions (such as intervention type, research population, and research conclusions), thereby providing systematic evidence support for decision makers (Li et al., 2021).

Currently, no evidence mapping study has assessed and presented the effectiveness and adverse effects on TCM adjuvant therapy for severe pneumonia. Therefore, we use evidence mapping to comprehensively present the quality of evidence on TCM adjuvant therapy for severe pneumonia from the included RCTs, SRs, and meta-analyses, with the aim to provide reliable evidence for TCM efficacy and safety assessment and guidance for clinical application and future research.

## 2 Methods

### 2.1 Inclusion and exclusion criteria

All RCTs, SRs, and meta-analyses on TCM adjuvant therapies for severe pneumonia were included. Included RCTs were required to report the inclusion criteria of the participants. Participants had to be diagnosed with severe pneumonia according to the guidelines of the Respiratory Society of Chinese Medical Association or the Infectious Disease Society of America/American Thoracic Society, as well as other recognized and reliable reference standards such as the *Internal Medicine Journal.* There were no limitations on the gender or comorbidities of the patients, except they had to be 18 or older. Included TCM interventions were as follows: Chinese herbal injections, TCM decoctions or granules, Chinese patent medicine, acupuncture, Chinese massage, pulmonary rehabilitation, acupoint application, comprehensive nursing of Chinese medicine, etc. There were no restrictions on the mode of administration, dosage, or duration. Control measures were conventional treatment with or without placebo. No restrictions were imposed on blinding, language, or publication type. Animal experiments, descriptive studies, conference abstracts, case reports, reviews, clinical experiences, trial protocols, letters, editorials, and unavailable or duplicate publication articles were excluded. Novel coronavirus pneumonia (COVID-19) was excluded in this scoping study to ensure homogeneity.

### 2.2 Search strategy

We comprehensively searched literature published on PubMed, EMBASE, Cochrane Library, Web of Science, and four major Chinese electronic databases, including China National Knowledge Infrastructure (CNKI), Wanfang Data, Chinese Scientific Journals Database (CQVIP), and BioMedical Literature Database (SinoMed), from inception to August 2023.

Search strategies were constructed using combinations of words describing the population/disease of interest (“severe pneumonia” or “severe pulmonary inflammation” or “severe pulmonary infection” or “severe community acquired pneumonia” or “severe hospital acquired pneumonia”), and the studied concept (“Chinese medicine” or “Chinese patent medicine” or “Chinese and western medicine” or “TCM appropriate techniques” or “external treatment” or “acupuncture” or “electroacupuncture” or “moxibustion” or “magnetic therapy” or “massage” or “ear point” or “ear acupuncture” or “medicated bath” or “foot bath” or “acupoint application” or “cupping” or “meridian external counterpulsation” or “acupoint injection” or “fumigation of Chinese medicine” or “hot pack” or “iontophoresis” or “bloodletting” or “pulmonary rehabilitation”). Depending on characteristics of the database, medical subject headings (MeSH) and/or free vocabulary words were combined. The detailed search strategies for all databases are reported in Supplementary Material S1.

### 2.3 Study selection and data extraction

Management of search results and deduplication was performed by EndNote X9 (Clavirate Analytics, Spring Garden, PA, United States) software. Two reviewers (GS and JH) independently screened all potentially relevant studies based on recorded titles and abstracts and then cross-checked. In case of disagreement, the study was tentatively included for more information. Once a preliminary selection decision was made, the full texts of the selected studies were downloaded for further identification. A new selection process was then independently conducted by two reviewers (GS and JH) based on full-text review for eligible RCTs, SRs, or meta-analyses. A consensus meeting was organized with all researchers to make a final decision on divergent related studies.

### 2.4 Quality assessment

#### 2.4.1 Risk of bias assessment of the included RCTs

The *Cochrane Handbook* ROB tool (Cumpston et al., 2019) was used to assess the risk of bias of the included 354 RCTs in terms of the following seven items: random sequence generation, allocation concealment, blinding of participants and personnel, blinding of outcome assessment, incomplete outcome data, selective reporting, and other bias (including sample size calculation, baseline balance, and interest conflicts) (Cumpston et al., 2019). Each item was classified as “yes” (low risk of bias), “unclear” (unclear risk of bias), or “no” (high risk of bias).

#### 2.4.2 Methodological quality assessment of the included SRs and meta-analyses

The Assessment of Multiple Systematic Reviews 2 (AMSTAR-2) tool (Shea et al., 2017) was used to assess the risk of bias for included SRs or meta-analyses by two reviewers (JW and KX) independently. Disagreements were resolved until a consensus was reached by mutual discussion with a third reviewer (XK). AMSTAR-2 contains 16 items, each of which was evaluated with “Yes,” “Partial yes,” or “No,” and overall confidence according to the weaknesses in critical domains (items 2, 4, 7, 9, 11, 13, and 15) was divided into four levels: high, moderate, low, or critically low (Shea et al., 2017). In other words, there were four categories in the overall assessment results of SRs or meta-analyses: no or one non-critical weakness was rated as “High,” “Moderate” meant more than one non-critical weakness, “Low” was one critical flaw with or without non-critical weaknesses, and “Critically Low” was defined as more than one critical flaw with or without non-critical weaknesses.

#### 2.4.3 Risk of bias assessment of the included SRs or meta-analyses

Two reviewers (DS and JM) separately assessed the risk of bias for the included SRs or meta-analyses by the Risk of Bias in Systematic Review (ROBIS) tool (Whiting et al., 2016). Any disagreements were settled by discussion or consulting the third reviewer (XK). The ROBIS tool assessment was conducted in three phases, namely, assessing the relevance, identifying concerns about bias during the review process, and judging risk of bias. Phase 2 includes the following four domains: 1) study eligibility criteria, 2) identification and selection of studies, 3) data collection and study appraisal, and 4) synthesis and findings. Each domain comprised five or six questions with answers of “yes,” “probably yes,” “probably no,” “no,” or “no information.” The risk of bias in the domain was rated “Low Risk” if all answers were “yes” or “probably yes.” If the answers were “no” or “probably no,” that domain was considered as “High Risk,” and the remaining domains were considered as “Unclear Risk.” The risk of bias in this domain was ultimately classified into “Low Risk,” “High Risk,” or “Unclear Risk” (Whiting et al., 2016).

#### 2.4.4 Evidence quality assessment of outcomes for the included SRs or meta-analyses

Assessment of the evidence quality using the Grading of Recommendations Assessment, Development, and Evaluation (GRADE) system (Hultcrantz et al., 2020) was carried out by two reviewers (GS and JH) back-to-back. In case of disagreement, two reviewers (GS and JH) resolved any issues by discussion. If RCTs were included in SRs or meta-analyses, the result was initially identified with high confidence. Conversely, in case of observational studies (OSs), the evidence quality was low confidence. Five downgrade factors were then considered, including inconsistency, risk of bias, indirectness, imprecision, and publication bias, along with three upgrade factors, including larger effects, dose–response gradients, and plausible confounding. The overall quality of evidence was categorized as “High,” “Moderate,” “Low,” or “Very low.”

### 2.5 Data synthesis and analysis

An in-depth discussion was conducted. Five categories were divided according to both results and conclusions of the included SRs or meta-analyses. “Beneficial” meant that the conclusions and results reported clear beneficial effects without major concerns about supporting evidence; “Probably beneficial” indicated that the conclusions did not claim an actual benefit despite reporting a positive treatment effect, or the conclusions reported a potential benefit despite the result showing no significant difference; “no effect” showed that the conclusions and results provided evidence of no differences between intervention and comparison; “inconclusive” indicated that the study results were insufficient for the authors to conclude whether the intervention has a definitive or potential effect; and “harmful” suggested that the conclusions and results were reported to be a harmful effect. In addition, the main judgment indicators were long-term prognosis, clinical symptom outcomes, laboratory inflammation indicators, adverse event description, and quality of life. A bubble plot was designed to display information in four dimensions (Ballesteros et al., 2017; Miake-Lye et al., 2019).

## 3 Results

### 3.1 Literature selection

**FIGURE 1:**
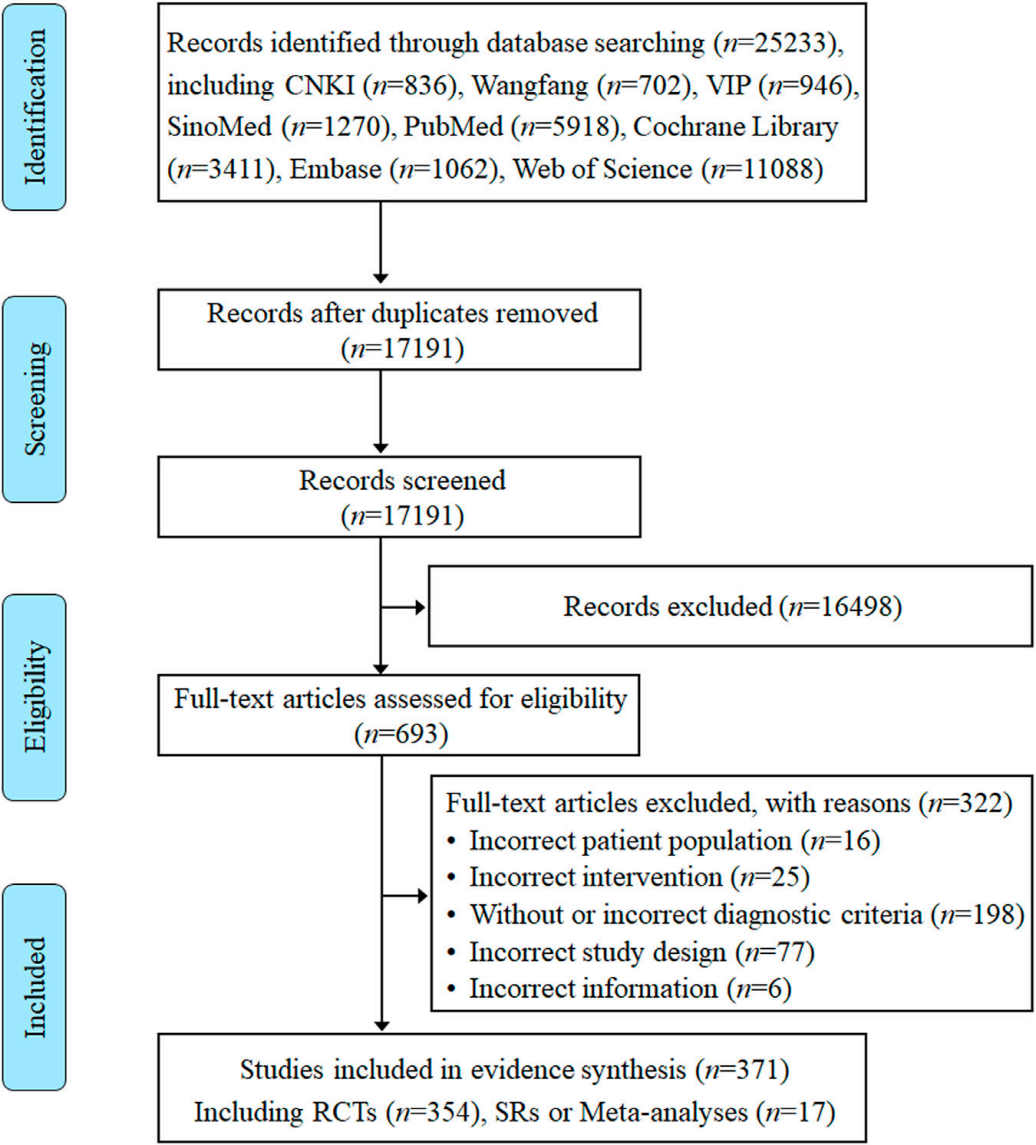
Flow diagram of the literature reviewing process and results.

Eight electronic database searches from inception to August 2023 yielded a total of 25,233 records. After removing duplicates, 17,191 records were screened according to titles and abstracts. The initial screening generated 693 studies identified as potentially relevant that were assessed against eligibility criteria. Of the remaining 693 articles, 322 articles were excluded after a detailed reading of the full text for not meeting the eligibility criteria. Ultimately, a total of 371 RCTs, SRs, and meta-analyses on TCM adjuvant therapy for severe pneumonia were included for systematic scoping review and evidence synthesis, of which 17 studies were SRs or meta-analyses. All detailed review processes, numbers of excluded, and reasons for full-text exclusions are shown in Figure 1.

### 3.2 Study characteristics

#### 3.2.1 Annual trends in publications

**FIGURE 2:**
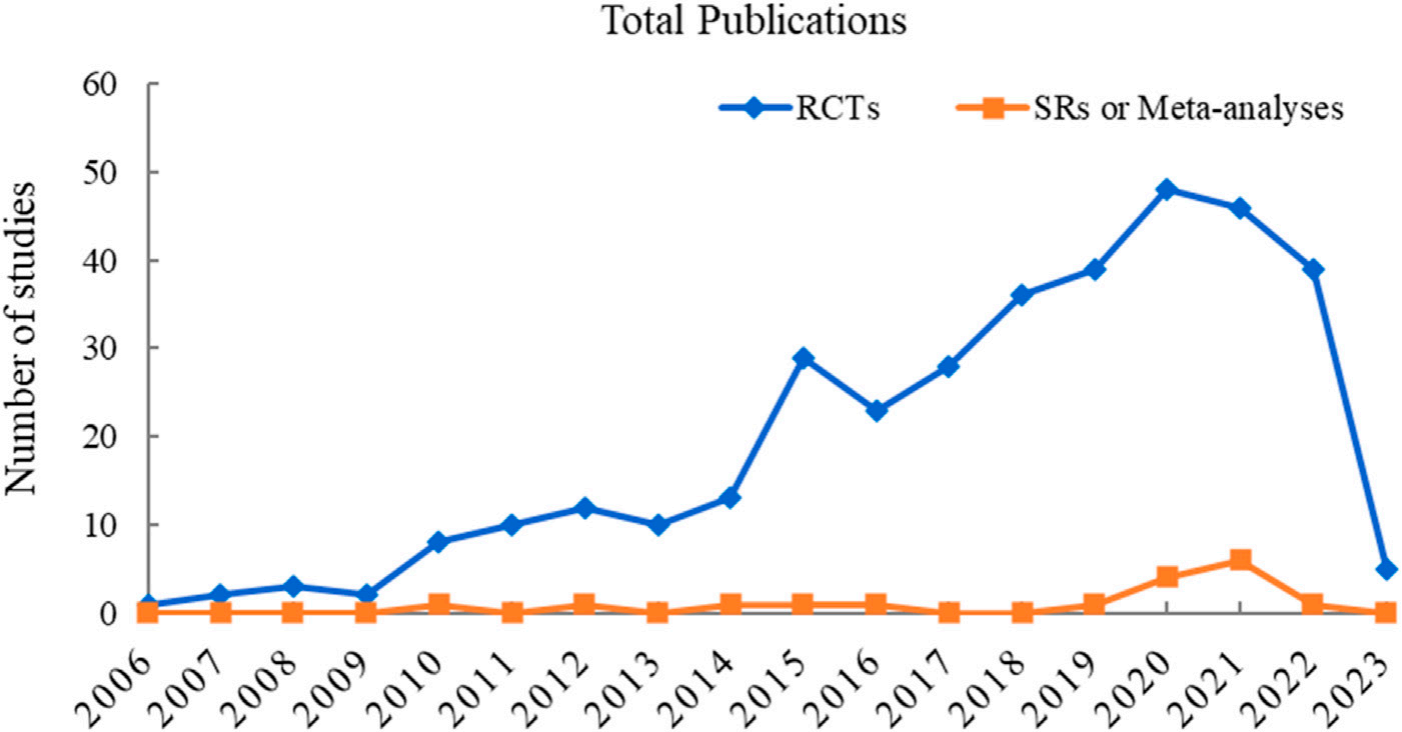
Annual trends in publications on RCTs and SRs or meta-analyses on TCM adjuvant therapy for severe pneumonia.

To visualize the trend of published studies on TCM adjuvant therapy for severe pneumonia over time, the association between the number of studies and the year of publication was plotted. RCTs on TCM adjuvant therapy for severe pneumonia first appeared in 2006, and SRs appeared in 2010, although the search began with the dates of the databases’ construction. However, there has been a progressive increase in the literature on TCM adjuvant therapy for severe pneumonia worldwide, peaking in 2020. The trend in publication of studies is shown in Figure 2.

#### 3.2.2 Geographical distribution

**FIGURE 3:**
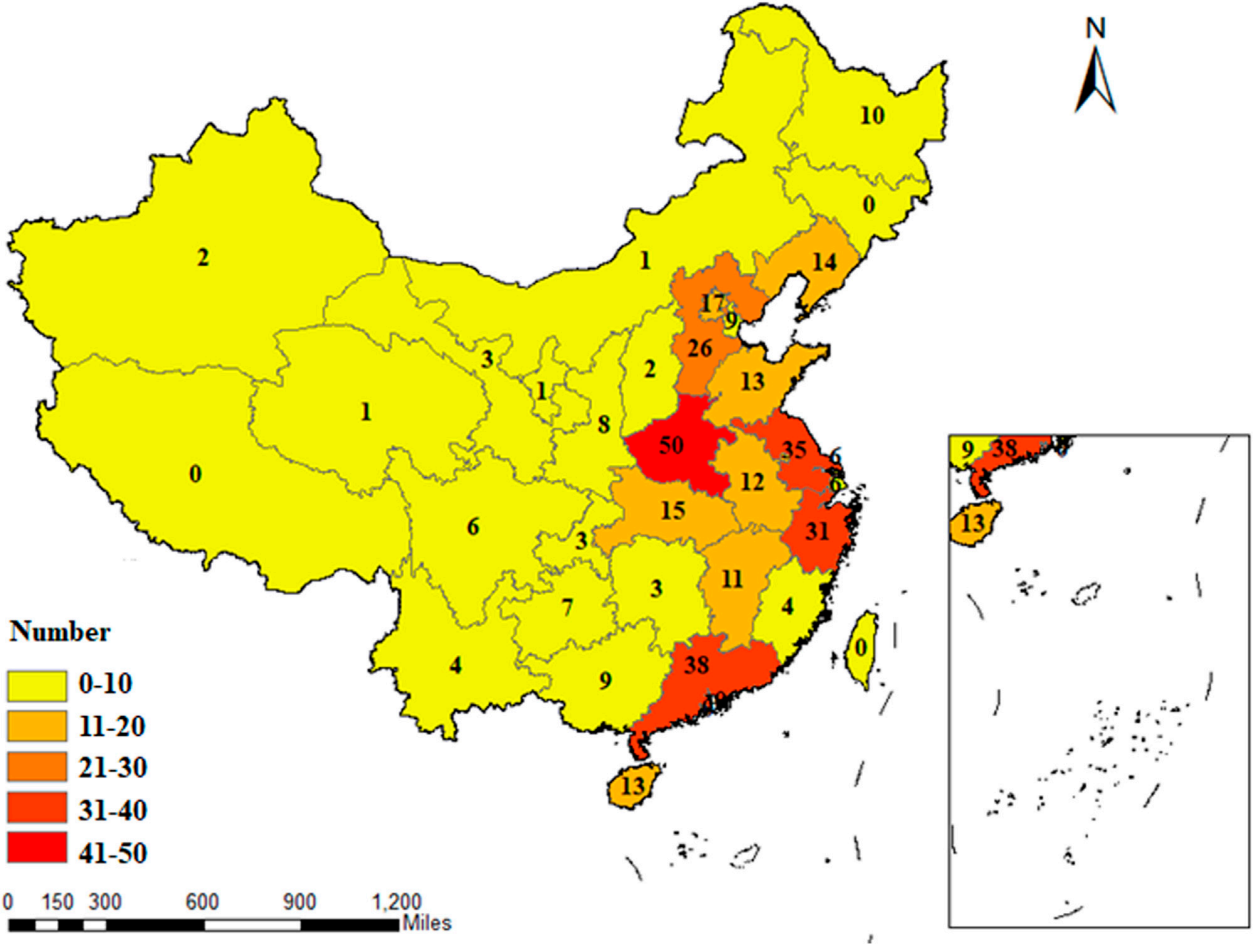
Geographical distribution of the primary investigators of RCTs on TCM adjuvant therapy for severe pneumonia.

The 354 RCTs considered were all conducted in China and located in 29 different provinces and municipalities. The largest number of RCTs were from Henan Province (*n* = 50), while no eligible published RCTs were found in Jilin Province, Tibet Autonomous Region, Hong Kong, Macao, and Taiwan Straits. The geographical distribution of RCTs on TCM adjuvant therapy for severe pneumonia for which there was eligible evidence was uneven, as shown in Figure 3.

#### 3.2.3 Intervention category distribution

**FIGURE 4:**
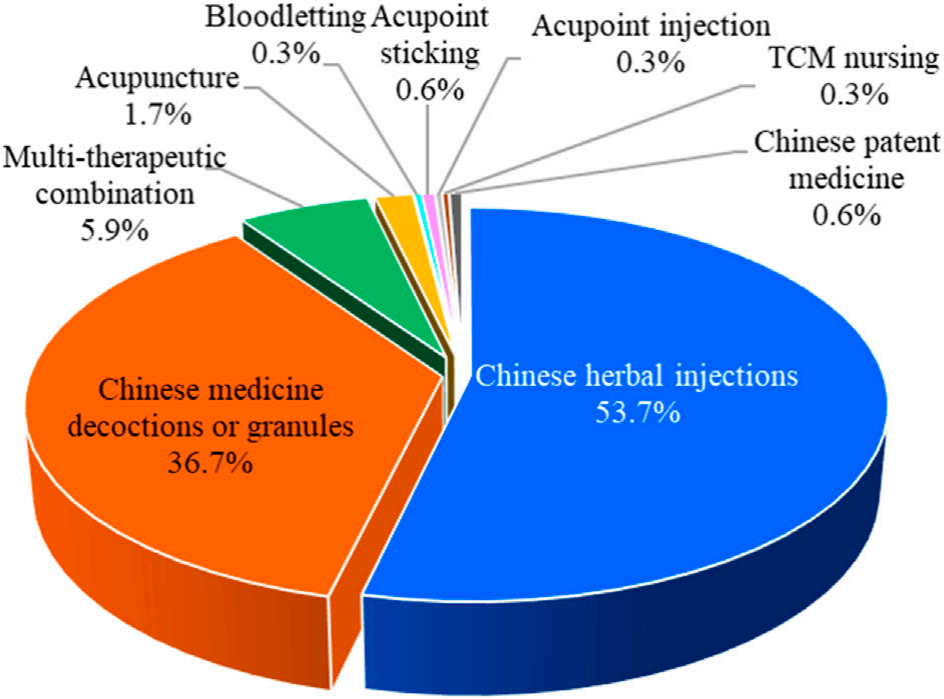
Proportion of different TCM intervention categories.

Chinese herbal injections, Chinese medicine decoctions or granules, bloodletting, acupoint sticking, acupuncture, acupoint injection, Chinese patent medicine, TCM nursing, and multitherapeutic combination are the nine major categories of TCM interventions. Among the 354 RCTs, there were 190 articles on Chinese herbal injections, followed by 130 articles on Chinese medicine decoctions or granules, 21 articles on multitherapeutic combinations, 6 articles on acupuncture, 2 articles on Chinese patent medicine and acupoint sticking, and 1 article each on bloodletting, acupoint injection, and TCM nursing. The proportion of different TCM intervention categories is shown in Figure 4.

Of the 354 RCTs, 190 (53.7%) assessed the efficacy of Chinese herbal injections for severe pneumonia, which include 122 studies of XueBiJing injection, 29 studies of TanReQing injection, 14 studies of ShenFu injection, 7 studies of XiYanPing injection, 7 studies of ReDuNing injection, 6 studies of ShenMai injection, 2 studies of DanShen injection, 2 studies of TanReQing combined with ShenFu injection, and 1 study of XingNaoJing injection. In addition, 72 RCTs included practitioner-made Chinese herbal experience decoctions. Furthermore, Qianjin Weijing decoction (14 studies), Maxing Shigan decoction (12 studies), and Xuanbai Chengqi decoction (9 studies) are classic TCM prescriptions that have been frequently studied in severe pneumonia.

#### 3.2.4 Study settings and sample sizes

Of the 354 RCTs, 348 RCTs were conducted in a single center, 1 RCT was a multicenter study with 33 centers, and 5 RCTs were not explicitly reported. The 354 RCTs included 30,113 participants; the sample size ranged from 30 to 675 per study, with an average of 85 participants. Most studies (269 RCTs, 76.0%) used a sample size of <100 patients, while 80 RCTs reported sample sizes between 100 and 200 patients, and only 5 RCTs reported sample sizes larger than 200 patients. The distribution of sample sizes is shown in Table 1.

**TABLE 1:** Clinical characteristics reported in the included RCTs on TCM adjuvant therapy for severe pneumonia.

Item	Details	N (%)	Item	Details	N (%)
Study setting	Single-center study	348 (98.3)		Respiratory failure	8 (2.3)
	Multicenter study	1 (0.3)		Stroke	6 (1.7)
	Not reported	5 (1.4)		Sepsis	6 (1.7)
Sample size	<100	269 (76.0)		ARDS	5 (1.4)
	100–200	80 (22.6)		Heart failure or myocardial injury	5 (1.4)
	>200	5 (1.4)		Postoperative esophageal cancer	1 (0.3)
TCM syndrome	Reported	119 (33.6)		Diaphragmatic dysfunction	1 (0.3)
	Not reported	235 (66.4)		AECOPD	1 (0.3)
Treatment duration	<7 days	14 (4.0)		Post-thoracic surgery	1 (0.3)
	7–13 days	166 (46.9)		Not reported	311 (87.9)
	14–20 days	147 (41.5)	Funding source	Government	88 (24.9)
	21–28 days	6 (1.7)		Multiple funding	5 (1.4)
	Not reported	21 (6.0)		Not reported	261 (73.7)
Comorbidity	Gastrointestinal dysfunction	9 (2.5)			

#### 3.2.5 TCM syndrome and treatment duration

TCM syndrome differentiation was explicitly reported in 119 RCTs, while the remaining 235 studies did not mention it. Treatment duration was clearly documented in 333 RCT studies (94.1%). A 14-day treatment duration was mostly frequently employed (140 studies), followed by a 7-day treatment duration (125 studies). The distribution of durations of TCM adjuvant therapy for severe pneumonia is shown in Table 1.

#### 3.2.6 Comorbidity

Severe pneumonia and its comorbidities was the study disease reported in 43 RCT studies. Among them, there were nine studies on comorbidities involving gastrointestinal dysfunction, eight studies on respiratory failure, six studies each on stroke and sepsis, and five studies each on acute respiratory distress syndrome (ARDS), heart failure or myocardial injury. Postoperative esophageal cancer, diaphragmatic dysfunction, acute exacerbation of chronic obstructive pulmonary disease (AECOPD), and post-thoracic surgery were comorbidities examined in one study each. The comorbidity details are shown in Table 1.

#### 3.2.7 Funding source

Of the 354 RCTs, 88 were supported by government funding sources, 5 were supported by multiple funding sources, and 261 did not report a funding source. The details are shown in Table 1.

### 3.3 Quality assessment

#### 3.3.1 Risk of bias for the 354 included RCTs

**FIGURE 5:**
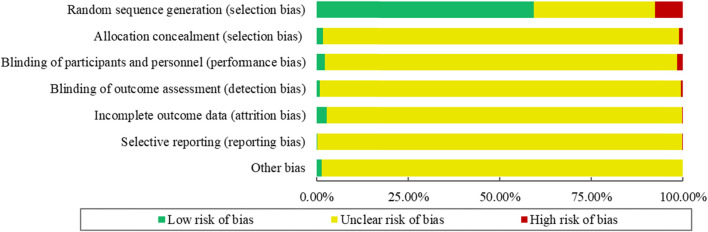
Summary of the risk of bias for RCTs.

The risk of bias for the 354 included RCTs as assessed by the *Cochrane Handbook* ROB tool. The results showed that 210 RCTs (59.32%) reported adequate random sequence generation, while 6 (1.69%) described an appropriate method of allocation concealment. Only 8 studies (2.26%) blinded the participants and personnel, and 3 RCTs (0.85%) used blinded outcome assessors. The risk of bias for incomplete outcome data was low for 10 RCTs (2.82%). Selective outcome reporting was regarded as low risk in only 1 RCT (0.28%). No RCTs were determined to have a “Low” outcome for “Risk of other bias.” The summary of the ROB of included RCTs is shown in Figure 5.

#### 3.3.2 Methodological quality of the included SRs or meta-analyses

**FIGURE 6:**
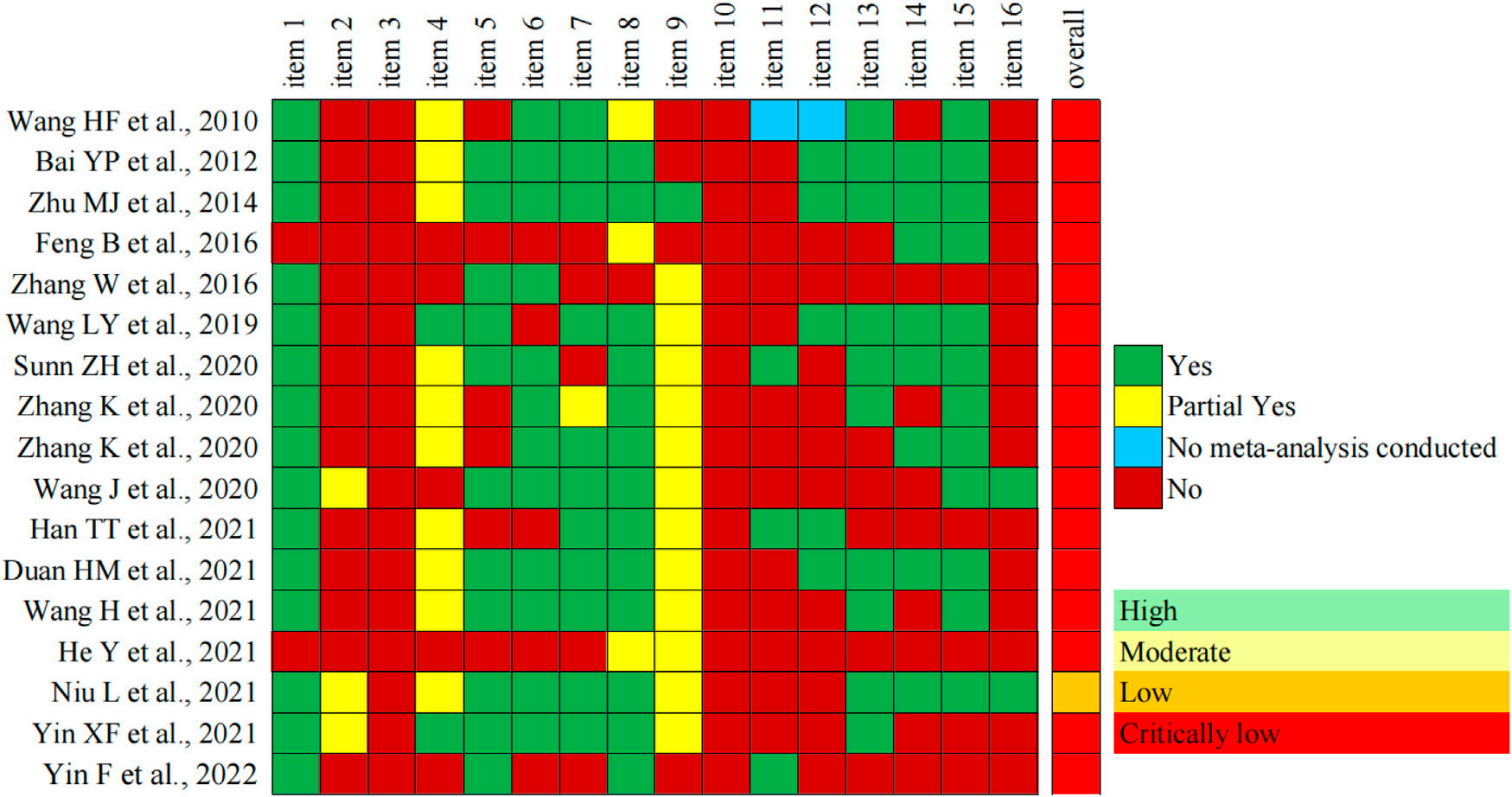
Methodological quality of the included SRs or meta-analyses.

In terms of methodological quality, 16 SRs or meta-analyses scored “Critically Low,” 1 SR scored “Low,” and no SR scored “Moderate” or “High” according to AMSTAR-2 criteria. The most frequent drawbacks were no mention of protocol, no reasonable explanation for selection of study design type for inclusion, no report on sources of funding for included studies, no investigation sources and adjustment for heterogeneity, and no statement for potential sources of interest conflict. The methodological quality of the included 17 SRs or meta-analyses is shown in Figure 6.

#### 3.3.3 Risk of bias of the 17 included SRs or meta-analyses

**FIGURE 7:**
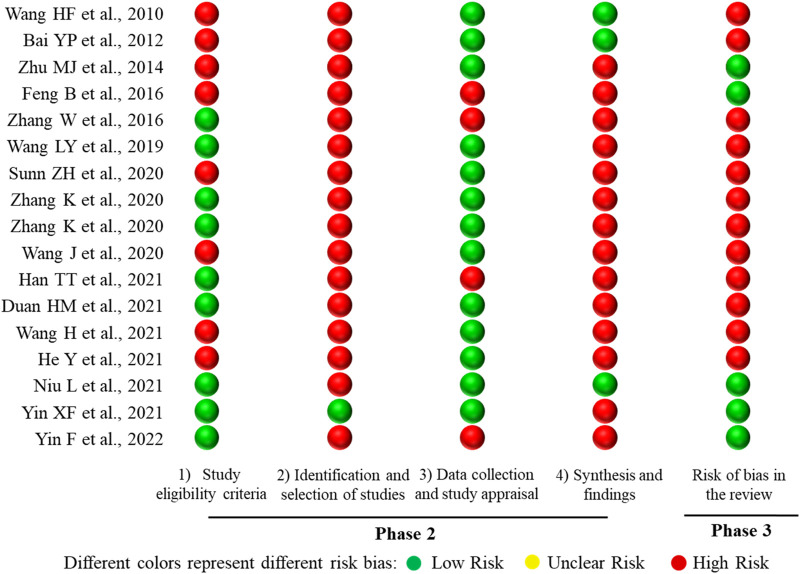
Mapping of the risk of bias of the 17 included SRs or meta-analyses. The lateral axis represented the ROBIS tool items, and the included SRs or meta-analyses are represented on the vertical axis.

The ROBIS tool includes three phases with four domains. All SRs or meta-analyses met the target question, and thus Phase 1 was rated as “Yes.” There are four domains in Phase 2. Domain 1 concerns study eligibility criteria, in which 8 SRs or meta-analyses (47.06%) were rated as “High Risk,” and 9 SRs or meta-analyses (52.94%) were assessed as “Low Risk.” Domain 2 addresses the identification and selection of studies; 16 SRs or meta-analyses (94.12%) were rated as “High Risk” and 1 SR (5.88%) was rated as “Low Risk.” For domain 3 regarding data collection and study appraisal, there were 4 SRs or meta-analyses (23.53%) rated as “High Risk” and 13 SRs or meta-analyses (76.47%) rated as “Low Risk.” With regard to domain 4—synthesis and findings—14 SRs or meta-analyses (82.35%) were rated as “High Risk,” and 3 SRs or meta-analyses (17.65%) were rated as “Low Risk.” Phase 3 considers the overall risk of bias of the 17 included SRs or meta-analyses, with 5 SRs or meta-analyses (29.41%) assessed as “Low Risk,” and 12 SRs or meta-analyses (70.59%) rated as “High Risk.” The results of risk of bias of the included SRs or meta-analyses are shown in Figure 7.

### 3.4 Evaluation of clinical efficacy of 354 included RCTs

The evaluation of the clinical efficacy of RCTs on TCM adjuvant therapy for severe pneumonia is divided into the following indicators: 1) efficacy outcomes: mortality, clinical total effective rate, mechanical ventilation time, ICU admission time, length of hospital stay, antibiotic used time, offline success rate, readmission rate, complication rate, and TCM syndrome efficacy; 2) clinical symptom outcomes: symptoms and signs (such as body temperature, heart rate, and respiratory rate), and symptoms or signs relief time; 3) laboratory outcomes: inflammatory indicators (including white blood cell counts (WBC), C-reactive protein (CRP) levels, procalcitonin (PCT), blood inflammatory factors, and bacteriological response), blood coagulation index (D-dimer and platelet counts), hemorheology, arterial blood gas indicators (including arterial oxygen pressure (PaO_2_), arterial oxygen saturation (SaO_2_), oxygen index (OI), and arterial partial pressure of carbon dioxide (PaCO_2_)), lung function (including forced vital capacity (FVC) and forced expiratory volume in the first second (FEV1)), and pulmonary imaging features; 4) life quality outcomes: Murray lung injury score, systemic inflammatory response syndrome (SIRS) score, Marshall score, pneumonia severity index (PSI) score, Acute Physiology and Chronic Health Evaluation (APACHE) II score, clinical pulmonary infection score (CPIS), and Sequential Organ Failure Assessment (SOFA) score; 5) safety outcomes; and 6) economic outcomes: e,g., hospitalization expenses.

Only three RCTs provided explicit primary outcome indicators. The main outcome indicators in one RCT were the critical care pain observation tool (CPOT) score, the Richmond agitation-sedation score (RASS), the reaching standard rate and time of shallow sedation and analgesia, the dosage of analgesic and sedative drugs, and the complication rate. These indicators were used to assess the analgesic and sedative effects of acupuncture on elderly patients with severe pneumonia on invasive mechanical ventilation. The main outcome indicators in the other two RCTs were the PSI improvement rate and the effective rate, respectively.

**FIGURE 8:**
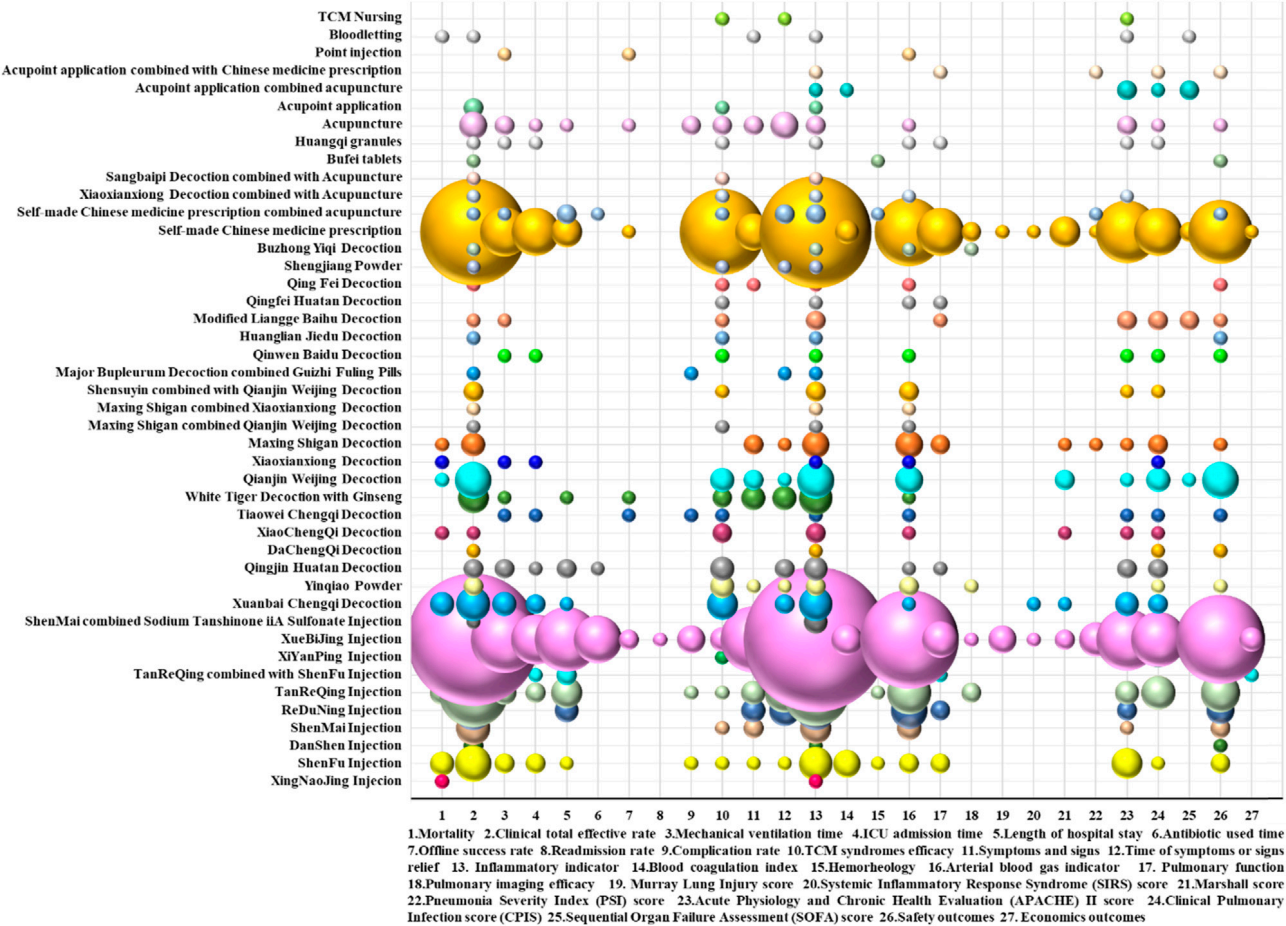
Evidence distribution of clinical efficacy evaluation on TCM adjuvant therapy for severe pneumonia. The size of the bubbles represents the numbers of included RCTs. Different colors represent various interventions. Efficacy indicators are represented on the lateral axis, and various intervention categories are shown on the vertical axis.

A bubble plot shows that the clinical RCTs on TCM adjuvant therapy for severe pneumonia paid more attention to inflammation indicators, clinical total effective rate, arterial blood gas indicators (PaO_2_, SaO_2_, OI, and PaCO_2_), safety indicators, time of symptoms or signs relief, and TCM syndromes efficacy and less attention to economic evaluation, hemorheology, pulmonary imaging features, the Murray score, the SIRS score, and the readmission rate. Evidence distribution of clinical efficacy evaluation on TCM adjuvant therapy for severe pneumonia is shown in Figure 8.

### 3.5 Evidence quality of outcomes of included 17 SRs or meta-analyses

**FIGURE 9:**
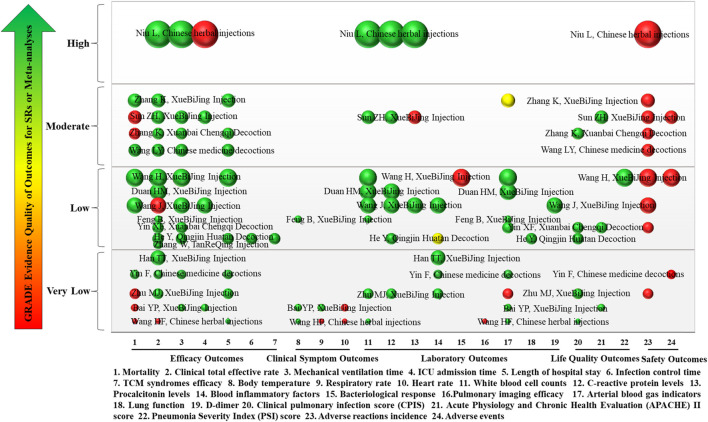
Mapping of evidence quality of efficacy, clinical signs, laboratory, life quality, and safety outcomes. The size of the bubbles represents the numbers of participants included in the SRs or meta-analyses. Different colors indicate statistical differences. Green represents *p* < 0.05, red indicates *p* > 0.05, and yellow represents mixed statistical significance. Outcomes are represented on the lateral axis, and the vertical axis indicates evidence of the quality of outcomes. The label on each bubble shows the first author and intervention of the included SRs or meta-analyses. Each horizontal row represents an SR or meta-analysis.

The types of studies included in the 17 SRs or meta-analyses were all RCTs. Overall, the quality of evidence for each quantitative synthesis outcome across the 17 included SRs or meta-analyses was assessed using the GRADE system, with most outcomes rated as “Low,” “Very Low,” or “Moderate” quality. Details of evidence quality of outcomes are shown in Figure 9.

#### 3.5.1 Efficacy outcomes

Eight efficacy outcomes were included in the 17 SRs or meta-analyses, including mortality, clinical total effective rate, mechanical ventilation time, length of ICU stay, length of hospital stay, infection control time, and TCM syndromes efficacy. Mortality was reported in 10 SRs or meta-analyses (58.82%), of which evidence quality was “Moderate” in 4, “Low” in 2, and “Very Low” in 4. Clinical total effective rate was reported in 16 SRs or meta-analyses (94.12%). The evidence quality was “High” in 1, “Moderate” in 4, “Low” in 7, and “Very Low” in 4. Mechanical ventilation time was reported in 10 SRs or meta-analyses (58.82%), of which evidence quality was “High” in 1, “Moderate” in 3, “Low” in 4, and “Very Low” in 2. Length of ICU stay was reported in 4 SRs or meta-analyses (25%) with 1 each of “High,” “Moderate,” “Low,” and “Very Low” evidence quality. Length of hospital stay was reported in 8 SRs or meta-analyses (47.06%); 3 were “Moderate” quality, 3 were “Very Low,” and 2 was “Low.” Infection control time and TCM syndromes efficacy were each reported in 1 SR (5.88%), and both had “Low” quality evidence.

The results showed that TCM combined with conventional treatment could improve the clinical total effective rate and TCM syndromes efficacy, as well as shorten mechanical ventilation time, infection control time, and length of hospital and ICU stay for patients with severe pneumonia compared with conventional treatment only (*p* < 0.05).

#### 3.5.2 Clinical symptom outcomes

There were three clinical symptom outcomes in the included 17 SRs or meta-analyses: temperature, respiratory rate, and heart rate. Temperature was reported in three SRs or meta-analyses (18.75%), of which evidence quality was “Low” in one and “Very Low” in two. Respiratory rate and heart rate were reported in two SRs or meta-analyses (12.5%), both of “Very Low” quality.

The results showed that, compared with conventional treatment only, TCM combined with conventional treatment could significantly reduce temperature, respiratory rate, and heart rate (*p* < 0.05).

#### 3.5.3 Laboratory outcomes

Nine laboratory outcomes were reported in the included 17 SRs or meta-analyses: WBC, CRP, PCT, blood inflammatory factors, bacteriological response, pulmonary imaging features, arterial blood gas indicators (PaO_2_, SaO_2_, OI, and PaCO_2_), lung function (FVC and FEV1), and D-dimer levels. WBC was reported in eight SRs or meta-analyses (47.06%), with one SR each of “High” and “Moderate” evidence quality, four of “Low,” and two of “Very Low.” Six SRs or meta-analyses (35.29%) reported CRP. The evidence quality was “High,” “Moderate,” and “Very Low” in one case each, and there were three cases of “Low” quality. PCT levels were reported in three SRs or meta-analyses (17.65%), one each of “High,” “Moderate,” and “Low” evidence quality. Blood inflammatory factors were reported in five SRs or meta-analyses (29.41%), with “Very Low” evidence quality in three and “Low” in two. There was only one SR (5.88%) each reported for bacteriological response, lung function (FVC and FEV1), and D-dimer levels, all of which were “Low” quality evidence. There was only one SR (5.88%) report on pulmonary imaging features, which was “Very Low” quality evidence. Arterial blood gas indicators (PaO_2_, SaO_2_, OI, and PaCO_2_) were reported in nine SRs or meta-analyses (52.94%), with one having “Moderate,” four “Low,” and four “Very Low” quality evidence.

The results showed that, compared with conventional treatment only, TCM combined with conventional treatment could significantly reduce WBC and levels of CRP, PCT, blood inflammatory factors, bacteriological response, and D-dimer; in addition, it could improve pulmonary imaging features, arterial blood gas indicators (PaO_2_, SaO_2_, OI, and PaCO_2_), and lung function (FVC and FEV1) (*p* < 0.05).

#### 3.5.4 Life quality outcomes

The 17 SRs or meta-analyses included three life quality outcomes: CPIS, APACHE II, and PSI score. CPIS score was reported in five SRs or meta-analyses (29.41%) with one “Moderate” and two each of “Low” and “Very Low.” APACHE II score was reported in three SRs or meta-analyses (17.65%) with one each of “Moderate,” “Low,” and “Very Low.” There was only one SR (5.88%) report on PSI score, which was “Low” quality evidence.

The results showed that TCM combined with conventional treatment could decrease CPIS, APACHE II score, and PSI score compared to conventional treatment only (*p* < 0.05).

#### 3.5.5 Safety outcomes

There were two safety outcomes in the included 17 SRs or meta-analyses: adverse reactions and incidence of adverse events. Adverse reactions were reported in 10 SRs or meta-analyses (58.82%), with 1 of “High” evidence quality, 4 of “Moderate,” 3 of “Low,” and 1 of “Very Low.”

The results showed no significant difference in adverse reactions and incidence of adverse events between TCM combined with conventional treatment and conventional treatment only (*p* > 0.05). The adverse reactions of XueBiJing injections included skin itching, dizziness, headache, nausea, diarrhea, rash, and upper limb pain. Common adverse events associated with TCM presented as gastrointestinal issues and headaches. There was a lack of reported adverse responses associated with the administration of Xuanbai Chengqi decoction.

### 3.6 Curative effect analysis of included 17 SRs or meta-analyses

**FIGURE 10:**
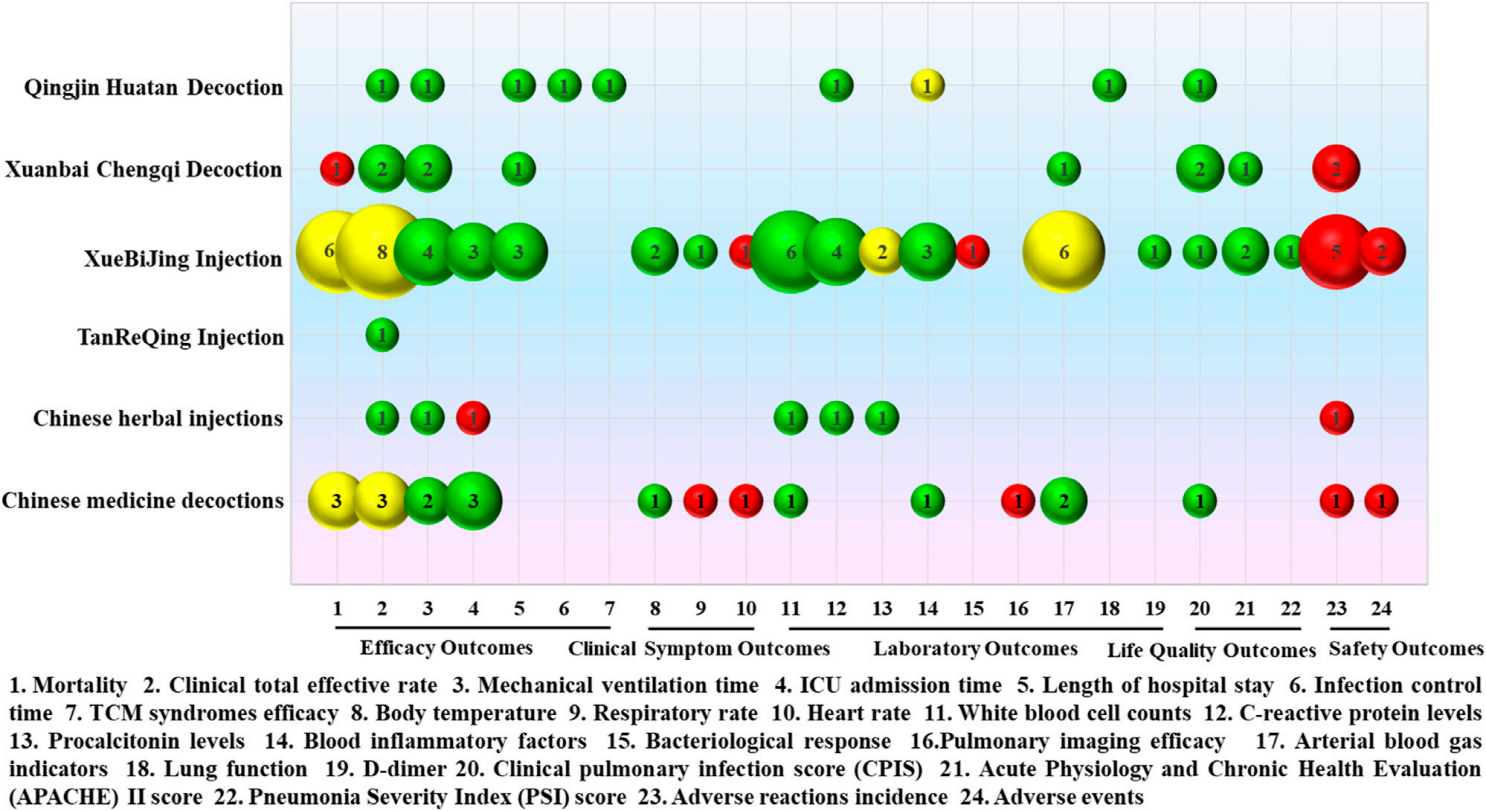
Mapping for the efficacy analysis of various intervention measures in the treatment of severe pneumonia with Chinese medicine. The size of the bubbles represents the numbers of participants included in the SRs or meta-analyses. Different colors indicate statistical differences. Green represents *p* < 0.05, red indicates *p* > 0.05, and yellow represents mixed statistical significance. Outcomes are represented on the lateral axis, and the vertical axis indicates the traditional Chinese medicine intervention.

The intervention measures with Chinese medicine in the treatment of severe pneumonia based on 17 SRs or meta-analyses can be roughly classified as Qingjin Huatan decoction, Xuanbai Chengqi decoction, XueBiJing injection, TanReQing injection, Chinese herbal injections, and Chinese medicine decoctions. Details of the curative effect analysis for various intervention measures based on 17 SRs or meta-analyses are shown in Figure 10.

A single meta-analysis was conducted to assess the effectiveness of Qingjin Huatan decoction in treating severe pneumonia compared to conventional treatment administered individually, which was of “Low” evidence quality. The results showed that Qingjin Huatan decoction combined with conventional treatment in the treatment of severe pneumonia decreased the clinical total effective rate (OR = 4.28, 95% CI: 1.94–9.47, *p* = 0.0003) and shortened the mechanical ventilation time (MD = −2.17, 95% CI: −2.34 ∼ −2.00, *p* < 0.00001), length of hospital stay (MD = −2.76, 95% CI: −4.55 ∼ −0.96, *p* = 0.003), and infection control time (MD = −2.72, 95% CI: −3.42 ∼ −2.03, *p* < 0.00001), decreased the TCM syndrome score (MD = −2.28, 95% CI: −2.90 ∼ −1.66, *p* < 0.00001) and levels of C-reactive protein (MD = −6.52, 95% CI: −7.38 ∼ −5.65, *p* < 0.00001), improved lung function, including forced expiratory volume in one second (FEV1, MD = 0.46, 95% CI: 0.40 ∼ 0.53, *p* < 0.00001), forced vital capacity (FVC, MD = 1.33, 95% CI: 1.11 ∼ 1.54, *p* < 0.00001), and peak expiratory flow (PEF, MD = 0.76, 95% CI: 0.65 ∼ 0.87, *p* < 0.00001). The clinical pulmonary infection score (CPIS, MD = −3.99, 95% CI: −6.61 ∼ −1.82, *p* = 0.00003) was better than that of conventional treatment alone.

Two SRs, which were rated as “Low” and “Moderate” quality of evidence, were conducted to evaluate the effectiveness of Xuanbai Chengqi decoction. The results showed that Xuanbai Chengqi decoction could reduce the clinical total effective rate of patients with severe pneumonia (RR = 1.26, 95% CI: 1.17–1.37, *p* < 0.00001; RR = 1.23, 95% CI: 1.16–1.30, *p* < 0.00001), and shorten the mechanical ventilation time (MD = −85.19, 95% CI: −152.06 ∼ −18.32, *p* = 0.01; MD = −101.41, 95% CI: −140.47 ∼ 62.34, *p* < 0.00001) and length of hospital stay (MD = −3.19, 95% CI: −5.64 ∼ −0.75, *p* = 0.01). Additionally, it was observed that Xuanbai Chengqi decoction led to a decrease in the CPIS score (MD = −2.08, 95% CI: −2.40 ∼ −1.76, *p* < 0.00001; MD = −2.02, 95% CI: −2.42 ∼ −1.63, *p* < 0.00001) and the Acute Physiology and Chronic Health Evaluation (APACHE) II score (MD = −6.81, 95% CI: −8.26–5.37, *p* < 0.00001), without any significant adverse reactions. However, the results did not show a statistically significant difference in the reduction of mortality (RR = 0.74, 95% CI: 0.29–1.87, *p* = 0.52).

Nine SRs or meta-analyses were conducted to evaluate the efficacy of XueBiJing injection, with evidence quality of “Very Low” for three, of “Low” for four, and “Moderate” for two. The results showed that XueBiJing injection could shorten mechanical ventilation time, ICU admission time, and length of hospital stay and reduce body temperature, respiratory rate, white blood cell counts, C-reactive protein, blood inflammatory factors, and D-dimer levels. Additionally, XueBiJing injection was found to have positive impacts on CPIS, the APACHE II score, and the pneumonia severity index (PSI) score. Uncertainties exist about the reduction of mortality, clinical total effective rate, procalcitonin levels, and arterial blood gas indicators. There were no statistically significant changes in heart rate and bacteriological response. Furthermore, there were no obvious adverse reactions.

There was only one “Low” quality evidence SR study on TanReQing injection. According to the findings, TanReQing injection might considerably lower the clinical total effective rate of severe pneumonia (OR = 0.31, 95% CI: 0.21 ∼ 0.47, *p* < 0.00001).

There was one network meta-analysis study on Chinese herbal injections, which was of “High” quality evidence. The findings of the SR indicate that the administration of Chinese herbal injections in patients with severe pneumonia resulted in favorable outcomes in terms of the clinical total effective rate, white blood cell counts, and C-reactive protein and procalcitonin levels, as well as a reduction in mechanical ventilation time compared to conventional treatment alone. Furthermore, no significant adverse reactions were observed. However, there was no statistical difference in the duration of ICU admission.

Three SRs or meta-analyses were conducted on Chinese medicine decoctions, with two showing “Very Low” and one showing “Moderate” quality evidence. The results showed that treating severe pneumonia with Chinese medicine decoctions could significantly shorten mechanical ventilation time and ICU admission time and reduce body temperature, white blood cell count, and blood cell inflammatory factors, as well as improve arterial blood gas indicators and CPIS scores. There were no statistically significant differences in respiratory rate, heart rate, and lung imaging efficacy. The statistical significance of reductions of mortality and total clinical effectiveness varied in different SRs or meta-analyses. In addition, there was no significant difference between Chinese medicine decoctions combined with conventional treatment and conventional treatment alone in reducing the incidence of adverse reactions and adverse events.

### 3.7 Specific findings from SRs or meta-analyses in the evidence mapping

**FIGURE 11:**
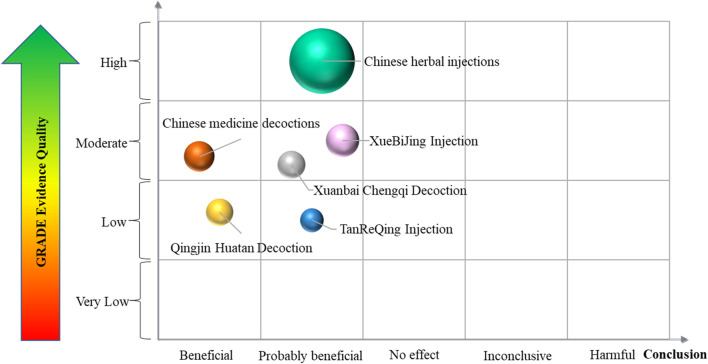
Evidence mapping on TCM adjuvant therapy for severe pneumonia. Each bubble represents a traditional Chinese medicine intervention. The size of the bubbles represents the numbers of participants included in the SRs or meta-analyses. The rating of conclusions is represented on the lateral axis, and the vertical axis indicates evidence quality.

The overall evidence quality of interventions that overlap was considered. Individual SRs reflected the conclusions, which were confirmed by an internal review. The evidence mapping on TCM adjuvant therapy for severe pneumonia is presented in Figure 11. Evidence mapping indicated that Qingjin Huatan decoction and Chinese medicine decoctions were mainly categorized with a “Beneficial” conclusion and may be a promising new target for severe pneumonia treatment. XueBiJing injection was the most studied of the targets and showed a majority of “Probably beneficial” treatment effects. In addition, Chinese herbal injections, Xuanbai Chengqi decoction, and TanReQing injection were also categorized as “Probably beneficial.” No SR or meta-analysis clearly declared that TCM adjuvant therapy was “harmful,” had “no effect,” or was “inconclusive” to severe pneumonia.

## 4 Discussion

### 4.1 Summary of the main findings

In this evidence-mapping study on TCM adjuvant therapy for severe pneumonia, we systematically searched the literature for relevant published RCTs and SRs or meta-analyses published until August 2023. Clinical studies data of 354 RCTs and 17 SRs or meta-analyses were comprehensively analyzed by words and graphs. We used the *Cochrane Handbook* ROB tool to assess the risk of bias in the included RCTs. The AMSTAR-2, the ROBIS tool, and the GRADE system were also used to assess the methodological quality, risk of bias, and evidence quality of the included SRs or meta-analyses. The distribution of evidence on TCM adjuvant therapy for severe pneumonia was then visually displayed.

RCT is the gold standard for assessing the efficacy and safety of interventions; 354 RCTs were included in this review. Most RCTs have given positive conclusions about the effectiveness and safety of TCM adjuvant therapy for severe pneumonia. However, some studies have problems such as inappropriate design, low quality of evidence, and limited international recognition of some outcome variables (such as total effective rate). Severe pneumonia, a critical disease always accompanied by respiratory failure, is often combined or complicated with multiple diseases, which presents certain difficulties and challenges in the conduction of clinical studies. We found that the earliest RCT on TCM adjuvant therapy for severe pneumonia appeared in 2006, and the first SR was published in 2010, which was far behind the research on the use of TCM with other diseases (Zhou et al., 2015; Kwan et al., 2019; Wang and Meng, 2021). Nonetheless, the important and exciting point is this: there has been a trend of increasing numbers of studies in recent years, not only for RCTs but also for SRs or meta-analyses. Currently, RCTs on TCM adjuvant therapy for severe pneumonia have been conducted in China, mainly with government funding, in 29 different provinces and municipalities, especially in Central, East and South China.

TCM is a systematic healthcare system developed from clinical experience based on a scientific, regulatory model. Originating approximately 5,000 years ago, unique practices of TCM include Chinese herbal medicine, dietetics, and other non-medication therapies such as acupuncture, massage, cupping therapy and Tai Chi (Matos et al., 2021). TCM is a science based on experience. Numerous classical TCM prescriptions with obvious curative effects and concise compatibility have been handed down to the present day and incorporated into ancient books as compulsory lessons for students dedicated to TCM.

In severe pneumonia, TCM adjuvant therapy approaches are extensive and diverse, with nine major categories dominated by Chinese herbal injections, especially Xuebijing injections, followed by Chinese medicine decoctions or granules. Relatively few studies investigated non-pharmacological therapies. In addition, the selection of TCM prescriptions was highly heterogeneous, with most being practitioner-made Chinese herbal experience decoctions, lacking research on classical TCM prescriptions, which may reflect the various academic ideas of different physicians. Sample size estimation is an essential step in clinical study conduction that must be planned properly to ensure the research time, personnel effort, and costs are not wasted. It is not uncommon that a study fails to explore even exaggerated treatment effects because of insufficient sample size (Wang and Ji, 2020). The sample size is limited despite the numerous RCTs on TCM adjuvant therapy for severe pneumonia. Most studies were conducted in a single center, and only five reported the sample size calculation, which might be due to the limitation of the actual research period or resources making it impossible to carry out multicenter large sample research. Only 93 RCTs were funded. In addition, there was no trial registration, which easily led to potential selective reporting bias. TCM syndrome differentiation is a distinctive feature and one of the fundamental theoretical systems of TCM. However, the study found that only 33.6% of RCTs reported TCM syndrome differentiation. Accurate syndrome differentiation is extremely important for the efficacy evaluation of TCM interventions, especially Chinese medicine decoctions, which might even limit the application and promotion of research results. Patients with severe pneumonia may experience very severe and long-lasting debility. The most commonly used treatment length was 14 days, followed by 7 days, which is consistent with the clinical disease characteristics. In addition, the quality of RCTs needs to be improved. Risk of bias assessment was performed on 354 RCTs, and the quality of the literature was found to be generally low, mainly due to a lack of information. Few RCTs mentioned assignment concealment, whether blinding methods were used, case drops or deaths, baseline balancing, conflicts of interest, and trial registration. Due to many methodological flaws, the results of RCTs are often biased. Inadequate reporting also leads to an inability to adequately assess the methodological quality of RCTs and limits their reproducibility (Glasziou et al., 2014). In this regard, the International Committee of Medical Journal Editors (ICMJE) proposed mandatory registration of RCTs, that is, detailed registration before the start of RCTs to make reporting more transparent and complete so as to achieve higher quality research (Vinkers et al., 2021). There were also some challenges with outcome indicators, such as unclear distinction between primary and secondary outcome indicators, lack of emphasis on the application of clinical endpoint indicators, inappropriate selection of alternative indicators, nonstandard evaluation criteria for TCM syndrome efficacy indicators, insufficient reports of safety events and economic evaluation, inaccurate description of composite indicators, and inconsistent reference standards.

RCTs are the primary source of evidence on the efficacy and safety of clinical interventions, while SRs, an essential part of evidence-based medicine, have been become the highest form of evidence as they synthesize all available evidence on a given topic (Murad et al., 2016). In addition, meta-analysis can give an overall effect estimate when combined with data. However, if the quality and criteria of SRs or meta-analyses vary widely, the findings of reviews may be exaggerated. The AMSTAR-2 and ROBIS are widely used as effective tools to evaluate the methodological quality and risk of bias of SRs or meta-analyses, respectively, with different characteristics and advantages in terms of assessment of such variation in standards (Perry et al., 2021). An increasing number of SRs or meta-analyses have been conducted on TCM adjuvant therapy for severe pneumonia. We used the AMSTAR-2 tool to assess methodological quality and to determine whether their conclusions were reliable. Unfortunately, we found that the overall confidence of the included reviews was generally “Critically Low.” The most frequent shortcomings were as follows: no mention of the protocol, no reasonable explanation for the selection of study design type for inclusion, no report on the funding sources for the included studies, no investigation sources and adjustment for heterogeneity, and no statement for potential sources of interest conflict. Therefore, researchers are advised to ensure the details of their study are fully documented before publication. Meanwhile, the assessment of the risk of bias using the ROBIS tool found that 29.41% of the SRs or meta-analyses were assessed as “Low Risk,” and 70.59% were assessed as “High Risk” for overall bias. As can be seen, improving the quality of studies is imperative.

In the past decade, the GRADE Working Group has developed tools and guidelines aimed at reducing unnecessary confusion and changes in assessing the certainty in comprehensive evidence (Hilton et al., 2021). GRADE quality reflects our confidence that the estimates of the effect are correct (Hultcrantz et al., 2017; Schünemann et al., 2022). The previous survey of stakeholders’ views and experiences confirmed that GRADE principles and processes are applicable to public health SRs (Rehfuess and Akl, 2013). According to the GRADE system, the evidence quality for outcomes of TCM combined with conventional treatment for severe pneumonia was mostly “Low” or “Moderate” confidence. Of the five downgrade factors, publication bias, risk of bias, and inconsistency were the common factors that lowered the level of evidence for SRs or meta-analyses on TCM adjuvant therapy for severe pneumonia. Major methodological flaws identified in this article include the presence of potential publication bias, not explicitly explaining the methods used for random sequence generation and assignment hiding, and not implementing a blind approach. Therefore, clinical researchers should register trial protocols before the start of a study and pay more attention to the details of the study design, which would help to produce high-quality RCTs. In addition, inconsistency was also an important degradation factor. Inconsistency refers to the degree of consistency among studies included in an SR, including clinical, methodological, and statistical inconsistency (Zhang et al., 2019). The GRADE system suggests that authors of SRs should generate and test a small number of priori assumptions related to patients, interventions, outcomes, and methods to explore the sources of heterogeneity and resolve inconsistency (Guyatt et al., 2011).

In terms of conclusion ratings, when comparing data between TCM combined with conventional treatment and conventional treatment only, most of the included SRs showed that TCM adjuvant therapy had a “Beneficial” or “Probably beneficial” conclusion, which indicated that TCM played a positive role in the treatment of severe pneumonia. In addition, data showed that XueBiJing injection was the most studied of the targets with a majority of “Probably beneficial” treatment effects, and Qingjin Huatan decoction was mainly categorized with a “Beneficial” conclusion and seemed to be a promising new target for severe pneumonia adjuvant treatment. As a result, it is reasonable to believe that TCM has some efficacy and safety benefits as an adjuvant therapy for severe pneumonia. More high-quality studies are needed to confirm this.

### 4.2 Strengths and limitations

Our study conducted a systematic and comprehensive search of eight databases and incorporated relatively reliable study designs, namely, RCTs, SRs and meta-analyses. Then, we used the AMSTAR-2 and ROBIS tools to assess the methodological quality and risk of bias of SRs or meta-analyses, and the GRADE system to assess the quality of evidence for inclusion in SR outcomes, visually presenting existing evidence results from multiple important dimensions in the form of bubble plots. In addition, we determined the rating of conclusions according to the description of both research results and conclusions, which may avoid the uncertainty caused by consulting only research results or conclusions (Li et al., 2021). Our study can be instructive for future research and avoid the waste of academic resources; it is also important for policymakers. Some limitations of this study should be mentioned. First, we did not include other study designs (such as case reports, cohort studies, or cross-sectional studies) because RCTs, SRs and meta-analyses generally provide the highest quality evidence for decision making. Second, only eight frequently used Chinese and English literature databases were searched. Literature from other sources, such as clinical trial registration websites, was not focused on, so there was an inevitable possibility of literature omission. Third, the methodological quality of the original studies was poor, which might affect the intrinsic authenticity of SRs or meta-analyses to some extent.

## 5 Conclusion

In summary, the published RCTs have some flaws, such as illogical design, limited sample size, insufficient attention paid to non-drug-therapy research and syndrome differentiation, improper selection or use of outcome indicators, and failure to provide high-quality evidence. Therefore, multicenter clinical studies with large sample sizes and high methodological quality are needed in the future. Most SRs or meta-analyses were rated as “Critically Low” confidence through the AMSTAR-2 tool and “High Risk” by the ROBIS tool. In addition, most outcomes were rated as low quality using the GRADE system. Overall, TCM combined with conventional medicine treatment may significantly improve efficacy, clinical signs, laboratory results, and quality of life outcomes in patients with severe pneumonia compared to conventional treatment only, with no difference in safety outcomes. Chinese medicine decoctions were primarily labeled as “Beneficial” in the treatment of severe pneumonia, among which the QingJin Huatan decoction is considered to be the most promising target, and the Xuanbai Chengqi decoction was also designated as “Probably beneficial.” The XueBiJing and TanReQing injections are two commonly used Chinese herbal injections for treating severe pneumonia, and both are thought to be “Probably beneficial.” Nevertheless, methodological design and quality must be improved in order to guide future clinical practice.

## Data Availability

The original contributions presented in the study are included in the article/Supplementary Material; further inquiries can be directed to the corresponding author.

## Abbreviations

AECOPD, acute exacerbation of chronic obstructive pulmonary disease; AMSTAR, the Assessment of Multiple Systematic Reviews; APACHE II score, Acute Physiology and Chronic Health Evaluation II score; ARDS, acute respiratory distress syndrome; COVID-19, novel coronavirus pneumonia; CRP, C-reactive protein; FEV1, forced expiratory volume in the first second; FVC, forced vital capacity; GRADE, the Grading of Recommendations Assessment, Development, and Evaluation; OI, oxygen index; PaO_2_, arterial oxygen pressure; PaCO_2_, arterial partial pressure of carbon dioxide; PCT, procalcitonin; PSI, pneumonia severity index; RCTs, randomized controlled trials; SaO_2_, arterial oxygen saturation; SIRS, systemic inflammatory response syndrome; SOFA score, Sequential Organ Failure Assessment score; SRs, systematic reviews; TCM, traditional Chinese medicine; WBC, white blood cell counts.
