# Supplementary material for: Efficacy and safety of traditional Chinese medicine adjuvant therapy for severe pneumonia: evidence mapping of the randomized controlled trials, systematic reviews, and meta-analyses
Source: Front Pharmacol. 2023 Sep 29;14:1227436. doi: 10.3389/fphar.2023.1227436 (PMC10570726; doi:10.3389/fphar.2023.1227436)
Supplement: Supplementary file 1 [file Table1.docx]

**Supplementary Material 1. The search strategy of PubMed database**

| **#** | **Searches** |
| --- | --- |
| #1 | "Medicine, Chinese Traditional"[Mesh] |
| #2 | Chinese medicine[Title/Abstract] OR Chinese patent medicine[Title/Abstract] OR Chinese and Western Medicine[Title/Abstract] OR TCM Appropriate Techniques[Title/Abstract] OR non-drug[Title/Abstract] OR external treatment[Title/Abstract] OR acupuncture[Title/Abstract] OR Electroacupuncture[Title/Abstract] OR moxibustion[Title/Abstract] OR Magnetic therapy[Title/Abstract] OR massage[Title/Abstract] OR ear point[Title/Abstract] OR ear acupuncture[Title/Abstract] OR Medicated bath[Title/Abstract] OR foot bath[Title/Abstract] OR Acupoint application[Title/Abstract] OR cupping[Title/Abstract] OR meridian external counterpulsation[Title/Abstract] OR acupoint injection[Title/Abstract] OR Fumigation[Title/Abstract] OR hot pack[Title/Abstract] OR iontophoresis[Title/Abstract] OR bloodletting[Title/Abstract] |
| #3 | severe pneumonia[Title/Abstract] OR severe pulmonary inflammation[Title/Abstract] OR severe pulmonary infection[Title/Abstract] OR severe community acquired pneumonia[Title/Abstract] OR severe hospital acquired pneumonia[Title/Abstract] OR SP[Title/Abstract] OR SCAP[Title/Abstract] OR SHAP[Title/Abstract] |
| #4 | #1 OR #2 |
| #5 | #3 AND #4 |

**Supplementary Material 1. The search strategy of CNKI database**

| **#** | **Searches** |
| --- | --- |
| #1 | SU=‘重症肺炎 + 重症感染 + 严重肺部感染 + 重症肺部炎症 + 重症细菌性肺炎 + SP + SCAP + SHAP’ AND TKA=‘中医药 + 中成药 + 中西医 + 中医适宜技术 + 非药物 + 外治 + 针刺 + 电针 + 灸 + 磁疗 + 推拿 + 耳穴 + 耳针 + 药浴 + 足浴 + 沐足 + 穴位贴敷 + 拔罐 + 火罐 + 经穴体外反搏 + 穴位注射 + 熏洗 + 热奄包 + 离子导入 + 穴位放血 + 肺康复’ |
